# Effects of the MyBFF@school program on anthropometry and body composition among overweight and obese adolescent schoolchildren

**DOI:** 10.1186/s12889-024-20725-0

**Published:** 2025-01-09

**Authors:** Nur Zati Iwani Ahmad Kamil, Abdul Halim Mokhtar, Abqariyah Yahya, Fuziah Md. Zain, Rusidah Selamat, Zahari Ishak, Muhammad Yazid Jalaludin

**Affiliations:** 1 Department of Pediatrics, Faculty of Medicine, University of MalayaUniversiti Malaya, Kuala Lumpur, Wilayah Persekutuan Kuala Lumpur 50603 Malaysia; 2 Endocrine and Metabolic Unit, Institute for Medical Research, National Institute of Health (NIH), Ministry of Health Malaysia, Setia Alam, Malaysia; 3 Department of Sports Medicine, Faculty of Medicine, Universiti Malaya, Kuala Lumpur, Wilayah Persekutuan Kuala Lumpur 50603 Malaysia; 4 Faculty of Sports and Exercise Science, Universiti Malaya, Kuala Lumpur, 50603 Malaysia; 5 Department of Social and Preventive Medicine, Faculty of Medicine, Universiti Malaya, Kuala Lumpur, Wilayah Persekutuan Kuala Lumpur 50603 Malaysia; 6 Department of Pediatrics, Putrajaya Hospital, Ministry of Health Malaysia, Jalan P9, Pusat Pentadbiran Kerajaan Persekutuan Presint 7, Putrajaya, Wilayah Persekutuan Putrajaya 62250 Malaysia; 7 Nutrition Division, Ministry of Health Malaysia, Level 1, Block E3, Complex E, Federal Government Administrative Centre, Putrajaya, Wilayah Persekutuan Putrajaya 62590 Malaysia; 8 FOSSLA, UCSI University, Kuala Lumpur, Wilayah Persekutuan Kuala Lumpur 56000 Malaysia; 9 Department of Sports Medicine, Faculty of Medicine, Universiti Malaya, Kuala Lumpur, Wilayah Persekutuan Kuala Lumpur 50603 Malaysia

**Keywords:** Obesity, Intervention, Schoolchildren, Effect, BMI *z*-score, Anthropometry, Percentage body fat, Skeletal muscle mass

## Abstract

**Background:**

Effective and feasible large-scale interventions are urgently needed to reverse the current rise in childhood obesity. The objective of this study was to evaluate the efficacy of a multicomponent intervention program, MyBFF@school, on anthropometric indices and body composition metrics among overweight and obese adolescent schoolchildren in Malaysia.

**Methods:**

This is a cluster randomized controlled trial which involved schoolchildren aged 13, 14 and 16 years old from 15 out of 415 government secondary schools in central Peninsular Malaysia which were randomly assigned into six intervention (*N* = 579 schoolchildren) and nine control (*N* = 462 schoolchildren).The intervention group followed MyBFF@school program carried out by trained personnel for 6 month while the control group only followed the existing school curriculum by the Ministry of Education. The primary outcomes presented in this study were body mass index adjusted for age (BMI *z*-score), waist circumference (WC), percentage body fat (PBF) and skeletal muscle mass (SMM), measured at baseline, three and six months. Analyses of all outcomes except for the baseline characteristics were conducted according to the intention-to-treat principle. Mixed linear models adjusted for baseline outcome value and gender were used to evaluate the effectiveness after three and six months of intervention.

**Results:**

Overall, there was no significant difference in the mean difference (MD) of BMI z-score (MD = 0.05, Confident Interval (95%CI: -0.077 to 0.194), WC (MD = 0.437, (95%CI:-3.64 to 0.892), PBF (MD = 0.977,95%CI:-1.04 to 3.0) and SMM (MD = 0.615,95%CI:-2.14,0.91) between the intervention and control group after 6 months of intervention after controlling for outcomes measured at baseline and gender.

**Conclusions:**

Although the MyBFF@school programme appeared promising in engaging children and promoting awareness of healthy behaviors, it did not lead to significant improvements in the anthropometric outcomes. Possible reasons for the lack of effectiveness could include the need for more intensive or targeted interventions, parental involvement, or challenges in sustaining behavior changes outside of school settings.

**Trial registration:**

Clinical trial number: NCT04155255, November 7, 2019 (Retrospective registered). National Medical Research Register: NMRR-13-439-16,563. Registered July 23, 2013. The intervention program was approved by the Medical Research and Ethics Committee (MREC), Ministry of Health Malaysia and Educational Planning and Research Division (EPRD), Ministry of Education Malaysia. It was funded by the Ministry of Health Malaysia.

## Background

Obesity is now recognized by the American Medical Association as a major global health concern [1]. It has been reported by the World Health Organization in 2016 that approximately 340 million children aged 5–18 years are overweight or obese [2]. These children and adolescents are at a greater risk of type 2 diabetes and metabolic syndrome, especially females, who are at a greater risk of early puberty and menarche [3] which may lead to irregular menstrual cycles and other hormonal issues, affecting overall health [4]. Obese children are at a markedly great risk of remaining obese throughout adulthood [5]. Diet, physical inactivity, psychological traits, and socioeconomic status are well-established modifiable factors influencing the risk of childhood obesity [6]. Most obesity prevention and intervention studies are conducted in the school setting [7], and regular interactions between children and teachers are considered an important factor contributing to the effectiveness of this school-based intervention/prevention approach [8]. In Malaysia, the National Health and Morbidity Survey reported in the Adolescents Health Survey 2022 that approximately 30.5% of Malaysian adolescents were either overweight or obese [9].

School-aged adolescents are physiologically and psychologically distinct from younger children and adults; therefore, there is a need for intervention programs tailored specifically for adolescents. For example, compared to younger children, adolescents may have or desire greater independence concerning their lifestyle and goal setting. Thus, motivation to change is mainly intrinsic. On the other hand, younger children are keener to follow instructions by parents and teachers [10]. It has also been shown that parental modeling of healthy eating and activity behavior is not as effective for supporting weight-loss among adolescents compared to younger children [11]. However, only a few intervention studies have targeted adolescents [12], and the results are inconsistent [13, 14]. Given the aforementioned rise in the prevalence of obesity among adolescents, there is a growing need for additional studies on obesity interventions targeting this age group.

Few obesity intervention studies involving obese children in Asia are available and have targeted mainly the primary schoolchildren [15]. In Malaysia, the majority have been small-sample studies (*n* = 50 ˗ 100) involving children age below 12 years old, focused on behavior change counseling [16–18]. The Malaysian Childhood Obesity Treatment (MASCOT) study was the pioneer of childhood obesity intervention study in Malaysia. However, MASCOT was a small scale study involving only 107 children aged 7–11 years in which only 50 children were allocated to follow the intervention program [16]. Furthermore, the MASCOT was a family-centred group treatment program which was directed to parents. Additionally, no significant difference was seen in the between group change in mean BMI *z*-score at six months’ intervention (*p* = 0.79), difference -0.04 (95% CI -0.33, 0.25). Thus, there is a need to develop a holistic childhood obesity intervention approach beyond parental involvement. The benefits of multimodal strategies, involving nutritional as well as physical exercise interventions in obese children, have been reported in many reviews and meta-analyses [19–21]. Subsequently, several other studies emerged but still either focusing children below the age of 12 years old [22, 23] or not targeted to children with obesity [24, 25].

To our knowledge, no obesity intervention study in Malaysia has included adolescents with obesity aged 13–17 years old. Additionally, none of the obesity intervention studies in Malaysia have integrated all three components of physical activity, nutritional education, and psychological module specifically for children and adolescents with obesity. Having said this, detailed protocol and intervention study justification was described in Mokhtar et al. [26]. Therefore, the MyBFF@school program was designed as a multicomponent intervention comprising physical activity, nutritional education, and psychological development specifically for overweight and obese primary schoolchildren (aged 9–11) and secondary school adolescents (aged 13–16) in Malaysia. For the purpose of this article, adolescent schoolchildren was used to represent this secondary school adolescents group. We evaluated the efficacy of a six-month MyBFF@school obesity intervention program on anthropometric measures and body composition in overweight and obese adolescent schoolchildren.

## Methods

### Study design and population

**Fig. 1:**
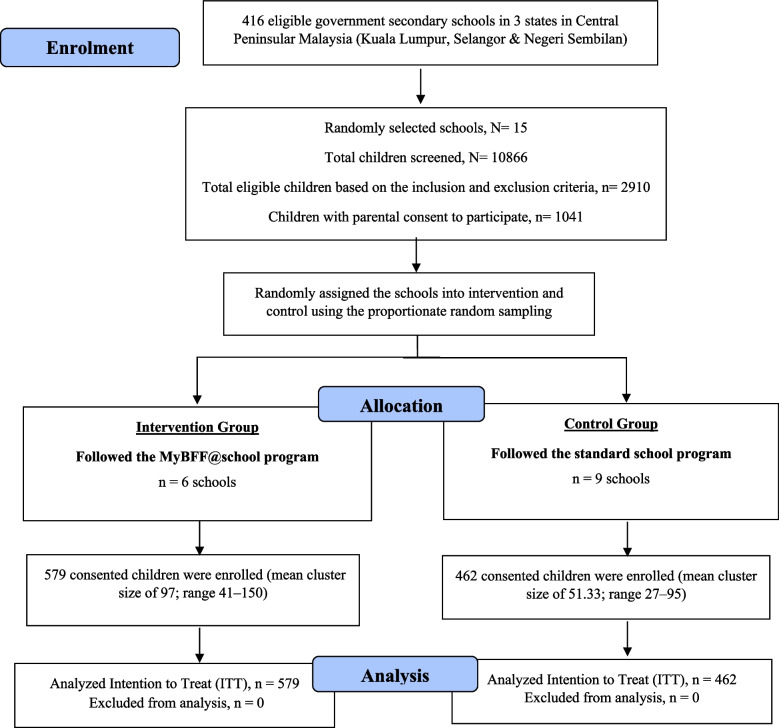
CONSORT diagram for central component in MyBFF@school

This study was a school-based cluster randomized controlled trial (C-RCT) (Fig. 1). The clusters were government secondary schools with schoolchildren aged 13, 14, and 16 years in three states in central Peninsular Malaysia, namely Kuala Lumpur, Selangor and Negeri Sembilan. A total of 15 out of 416 eligible government secondary schools were randomly assigned into six intervention (*n* = 579 children) and nine control schools (*n* = 462 children) taking into consideration the school type and location. The location of the schools as either in urban or rural areas was based on the current classification used by the Ministry of Education Malaysia which was adopted from the National Census by the Department of Statistics Malaysia (DOSM) 2010 [27]. Blinding of the intervention was not possible as the schools involved in the intervention or control needed to be disclosed for approval from the Ministry of Education, Malaysia apart from the staff in charge of the intervention schools needed to be trained. Recruitment of the participants within the selected clusters was based on the inclusion and exclusion criteria. The detailed methodology including the inclusion and exclusion was published by Mokhtar et al. [26]. Written informed consent was obtained from parents or guardians prior to the study. Schools selected for intervention underwent the MyBFF@school program, whereas control schools followed only the regular school health education syllabus. Schools involved in other obesity intervention programs were excluded from the study [26].

### MyBFF@school intervention package

MyBFF@school intervention package is described in another article in this supplement [26]. Briefly, MyBFF@school program is a multi-faceted obesity intervention incorporating physical activity in the form of small-sided games (SSG), nutrition, and psychology components. SSG (football, handball, and fun games) sessions were conducted for 40 min as allocated in the school curriculum. Two sessions were organized per week; therefore, a total of 80 min per week were spent on SSG activities. Nutrition education intervention (NEI) utilized the nutrition education modules (NEM) adapted from the Malaysian Childhood Obesity Treatment Trial (MASCOT)’s modules, was conducted on alternate week with psychology module for six months by trained research assistants (45–60 min per session). The main themes in the psychology modules were self-esteem, friendship, assertiveness, and positive thinking for a healthy lifestyle and stress management, guided by trained research assistants. Motivational talks and interactive activities were the two main mechanisms used to achieve the objectives, followed by a reflection session at the end of the activities. All components were carried out during physical education periods and co-curriculum activities in the school during school hours. The research team monitored the schoolchildren on a monthly basis, but formal assessment was performed at baseline, three and six months.

### Ethics statement

Ethics approval was granted by the Medical Research and Ethics Committee (MREC), Ministry of Health Malaysia (NMRR-13-439-16563). Written informed consent was obtained from parents or guardians, and every child was required to sign an assent form. All tests were performed in accordance with MREC approved guidelines.

### Anthropometric and body composition assessments

Prior to health and physical examinations, children were asked to fast overnight for at least 8 h. Anthropometric measurements were performed by trained personnel, and health examinations were performed by medical officers and pediatricians. Standing height was measured without shoes to the nearest 0.1 cm using a calibrated stadiometer (Seca 217; Seca, Hamburg, Germany). Body weight and body fat mass were measured in light clothing without shoes and socks to the nearest 0.1 kg using a precalibrated body impedance analyzer (InBody 720; InBody, Seoul, Korea). Waist circumference (WC) was measured twice to the nearest 0.1 cm over the skin midway between the tenth rib and the iliac crest at the end of normal expiration using a nonextensible tape (Seca 201; Seca, Hamburg, Germany), and the mean was recorded. Two readings of blood pressure were measured after 5 min of rest using a mercury sphygmomanometer (Accoson, Irvine, UK) in a seated position with the arm supported at the heart level, and the mean was recorded. Pubertal/sexual maturity was self-assessed following Tanner’s staging scale criteria [28, 29].

### Definition of measures

The BMI *z*-score was calculated using the WHO AnthroPlus 2007 software. Overweight, obese, and morbidly obese were defined by BMI *z* -scores above 1, 2, and 3 SDs, respectively, adjusted for age and gender according to the WHO BMI chart (2008) [30]. Abdominal obesity was defined as a WC of the 90th percentile or higher on the Malaysian WC chart [31].

### Adolescents

World Health Organization’s (WHO) define adolescents as those between the ages of 10 and 19 years [32]. In Malaysia schools were categorized as primary (age 7–12 years old) and secondary (age 13–17 years old). Therefore in this paper adolescents were referring to secondary school children age 13–16 years old participating in MyBFF@school program.

### Statistical analysis

All outcomes except for the baseline characteristics were analyzed according to the intention-to-treat principle and are reported at three and six months after the baseline. All data analyses were conducted using SPSS Statistics for Windows (Version 24; IBM Corp., Armonk, NY, USA) and Stata 2015 (StataCorp, College Station, TX, USA). Normality of continuous data was determined using the Kolmogorov–Smirnov test. Means and SDs were calculated for continuous variables, whereas enumeration variables are expressed as *n* (%). Continuous variables at baseline were compared between groups using independent samples *t*-tests, and categorical variables at baseline were compared using the chi-squared test. In this study we found 3 missing waist circumference measurement at baseline. Prior to imputing the missing values, missingness was investigated. We found the missing waist circumference was completely at random (MCAR). Thus multiple imputation was used to impute the missing values [33]. Within and between group change after month-3 and month-6 were obtained via mix linear model with the post intervention value for the outcomes as dependent variables (continuous variables) adjusted for baseline values and gender. The intracluster correlation coefficient (ICC) was also estimated.

## Results

### Characteristics of participants

At baseline a total of 1,041 adolescents (*n* = 579 in the intervention and *n* = 462 in the control group) participated in this study evaluating the MyBFF@school program, followed by 787 (*n* = 462 in the intervention and *n* = 325 in the control group) (75.6%) at month-3 and 730 (*n* = 385 in the intervention and *n* = 345 in the control group) (70.1%) at month-6 visits. Thus complete case analysis was used to describe the baseline characteristics of the intervention and control groups in Table 1 whereas the effect of the intervention at month-3 and month-6 was analyse using the intention to treat principle. The groups exhibited a comparable gender ratio, pubertal status, abdominal obesity rates (about 60%), and BMI $z$-score distributions (all $p>0.05$). The mean age was higher in the control group compared to the intervention group (14.3 ± 1.34 versus 14.1 ± 1.35 years). Looking at the ethnicity, majority of the participants in both group were of ethnic Malay, followed by Indian, Chinese and other minority ethnic.

**Table 1:** Baseline characteristics of adolescent participants in the MyBFF@school program

**Characteristic (** $n$**, %)**	**Intervention (579, 55.6%)**	**Control (462, 44.4%)**	**Χ**^**2**^	*p***-value**
**Mean age** (years ± SD)^a^	14.1 ± 1.35	14.3 ± 1.34		0.013
**Gender (**$n$**, %)**^b^				
Male	234 (40.4)	196 (42.4)	0.428	0.277
Female	345 (59.6)	266 (57.6)		
**Ethnicity (**$n$**, %)**^b^				
Malay	449 (78.2)	251 (70.7)	21.2	< 0.001
Chinese	31 (5.4)	27 (7.6)		
Indian	65 (11.3)	71 (20)		
Other	29 (5.1)	6 (1.7)		
**Pubertal status (**$n$**, %)**^b^				
Prepubertal	23 (4)	17 (3.7)	0.052	0.872
Pubertal (Tanner stage ≥ 2)	554 (96)	441 (96.3)		
**Abdominal obesity (**$n$**, %)**^b^				
WC < 90th centile	210 (36.4)	184 (39.9)	1.347	0.248
WC > 90th centile	367 (63.6)	277 (60.1)		
**BMI ** $z$**-score status (**$n$**, %)**^b^				
Overweight > + 1 SD	257 (44.4)	228 (49.4)	4.15	0.126
Obese > + 2 SD	247 (42.7)	190 (41.1)		
Morbidly obese > + 3 SD	75 (13)	44 (9.5)		

Missing data: 112 in ethnicity, 6 in pubertal status, and 3 in abdominal obesity

*WC* waist circumference, *SD* standard deviation

^a^Continuous variables at baseline were compared using independent samples *t*-tests (intervention versus control)

^b^Categorical variables at baseline were compared using the chi-squared test

### Effectiveness of intervention after three months

Outcomes adjusted for baseline are reported in Table 2. After three months, no significant within-group improvement was seen in all body composition outcomes neither in the intervention nor control group. Similarly no significant mean difference (MD) was seen in all parameters between intervention and control group.

**Table 2:** Adjusted within- and between-group differences in anthropometric and body composition indices (adjusted for baseline outcome value)

Body composition	Visit	Group	Change within-group	95%CI	Change between group (intervention v control)	95%CI	ICC
			**Mean Difference (SE)**		**Mean Difference (SE)**		
**BMI ** $z$**-score**	**Month 3**	Control	0.007 (0.052)	-0.096,0.110	-0.03(0.067)	-0.138,0.130	0.011^*^
		Intervention	− 0.070 (0.047)	-0.165,0.020			(0.002,0.038)
	**Month 6**	Control	-0.077 (0.051)	-0.177,0.002	0.050 (0.068)	-0.077,0.194	0.006
		Intervention	-0.094(0.040)	-0.191,0.004			(0.001,0.038)
**WC (cm)**	**Month 3**	Control	0.756(0.747)	-0.711,2.220	-1.380(1.150)	-3.64,0.892	0.017
		Intervention	-1.329(1.150)	-2.740,0.091			(0.006,0.047)
	**Month 6**	Control	0.347(0.789)	-1.210,1.900	0.437(1.180)	-1.956,1.759	0.013
		Intervention	0.257(0.750)	-1.240,1.750			(0.003,0.044)
**PBF(%)**	**Month 3**	Control	0.094(0.499)	-0.889,1.070	-0.440(0.940)	-2.290,1.410	0.041
		Intervention	-0.440(0.944)	-2.295,1.414			(0.017,0.094)
	**Month 6**	Control	-1.120(0.520)	-2.160,-0.880^*^	0.977(1.03)	-1.040,3.00	
		Intervention	-0.520(0.500)	-1.520,0.480			
**SMM (kg)**	**Month 3**	Control	0.104(0.360)	-0.566,0.774	-0.027(0.72)	-1.440,1.390	0.055
		Intervention	0.270(0.320)	-0.357,0.896			(0.024,0.122)
	**Month 6**	Control	0.804(0.360)	0.090,1.510^*^	-0.615 (0.770)	-2.140.0.190	0.060
		Intervention	0.390(0.310)	-0.218,1.00			(0.026,0.133)

For BMI z-scores,WC and PBF (%) negative changes indicate a reduction from baseline to month 3 and month 6. For SMM positive change indicate an increase from baseline to month 3 and month 6

*BMI* body mass index, *WC* waist circumference, *MD* mean difference, *SE* standard error, *SMM* skeletal muscle mass, *ICC* intracluster correlation coefficient

^*^p < 0.05

### Effectiveness of intervention after six months

The outcomes after six months were reported in Table 2. We found significant within group reduction of PBF (MD = -1.120%, 95%CI:-2.16 to-0.088) and increase of SMM (MD = 0.804 kg,95%CI:0.09–1.51) in the control group. However this improvement was not significant compared to the intervention group (MD = 0.977,95%CI:-1.04 to 3.0) and (MD = 0.615,95% CI: -2.14 to 0.91) respectively. Similar to month 3 no significant MD was seen in all other parameters between intervention and control group.

## Discussion

We developed MyBFF@school to address obesity and various sequelae in Malaysian schoolchildren. Evidence suggests that participation in interventions combining physical activity, dietary adjustments, and behavioral strategies can effectively help children who are obese achieve a healthier weight [34]. However, in this study we found no significant difference in the overall finding of the obesity related outcomes. Although it is not conclusive on what factors of lifestyle intervention programs in children and adolescents with obesity contribute to success in the context of improvement of selected obesity measures, studies have demonstrated a larger response of lifestyle interventions in children compared to adolescents [35–37] suggesting a new or different approach for obesity intervention in adolescents. In adolescents, other treatment options may be sought such as enhanced lifestyle interventions or additional medical and surgical interventions [38]. For example the use of gastrointestinal peptides (GLP 1) is approved for the treatment of obesity in those aged 12 years and older both in the USA and Europe [39]. Furthermore, in the recent 2023 American Academy of Pediatrics clinical practice guidelines bariatric surgery may be considered in adolescents ≥ 13 years of age with a BMI of ≥ 35 kg/m^2^ or 120% of the 95th percentile for age and sex, whichever is lower, as well as clinically significant disease, such as type 2 diabetes mellitus, non-alcoholic fatty liver disease, major orthopedic complications, obstructive sleep apnoea, the presence of cardiometabolic risk, or depressed quality of life [40].

Apart from that, easy access to unhealthy food such as fast food and sweetened beverages may be a contributing factor for the lack of improvement on the obesity measures. Moreover, adolescents have greater autonomy in their food choices compared to younger children. A study on fast-food consumption among young adolescents age 12–15-year-old reported that approximately 55.2% (51.3–59.1%) of the adolescents consumed fast food at least 1 day per week, and 10.3% (8.3–12.4%) did so 4–7 days per week [41]. However, in this study we only measured quantitative food frequency questionnaire (FFQ) for the past one week which was adopted for FFQ on fruit and vegetable from the WHO STEPwise Approach for Surveillance of Non-Communicable Diseases [42]. Hence the intake of micro and macro nutrient cannot be taken into consideration for adjustment in our model of analysis. Yet, our study highlights the need for effective obesity intervention in adolescents. This study add to the limited studies on the effect of obesity interventions in adolescents especially in Malaysia. Additionally MyBFF@school also include the psychological aspects to cater the psychosocial well being and reduce the impact of stigmatization. The findings from MyBFF@school will guide the decision-making process about intervention strategies to be implemented in adolescents with obesity. To the best of our knowledge, MyBFF@school was the first large scale multicomponent obesity intervention applied and evaluated for its effectiveness in overweight and obese children and adolescents studied in Malaysia.

## Conclusion

In summary, the MyBFF@school program demonstrated that a 6 month multicomponent intervention resulted in insignificant improvement of obesity measures in adolescents. We suggest that the obesity intervention program in adolescents to add further adaptations, addressing more potential out-of-school and external setting influences to be more successful.

## Data Availability

All relevant data are within the paper.

## Abbreviations

BMI: Body mass index
CI: Confidence interval
ICC: Intracluster correlation coefficient
MD: Mean difference
PBF: Percentage body fat
SD: Standard deviation
SMM: Skeletal muscle mass
WC: Waist circumference
WHO: World Health Organization
